# Pulp Reunion

**Published:** 1908-02-15

**Authors:** A. H. Fuller


### PULP REUNION.

BY A. H. FULLER, M. D., D. D. S.

In the February, 1899, number of *The Dental Review* (Vol. XIII, No. 2) an article was published with the following title: "Do the Pulps of Replanted Teeth Ever Reunite with the Tissues from Which They Have Been Separated."

The occasion for the article was a statement made before the New Jersey State Dental Society by Dr. Register, and published in *Items of Interest* of September, 1898, page 693. He is reported as saying, "I do not believe for a moment that under any conditions a tooth can be removed, replaced, and the circulation in the pulp re-established I think such a thing is absolutely impossible. '

In *The Dental Review* article, which was about four pages, I cited a number of cases reported and published in the dental journals that, if true (and without doubt they are true), would prove Dr. Register's belief to be incorrect and the statement misleading.

Within the past year it has been my good fortune to have had an opportunity to establish beyond a doubt the position I took that union is possible and does occur under favorable conditions and proper treatment.

I have the evidence of the patient and the patient's mother, also that of Dr. Frederick S. McKay, now of Colorado Springs, Colorado, who took charge of the case, and my own testimony to offer that the tooth was out of the mouth and on the floor, and can produce the patient himself to establish the present condition of the tooth.

One year ago Master Edwin H., ten years of age, while ascending steps leading to the school room, slipped, striking his face against an iron post, cutting his lip, knocking out the right upper temporary cuspid, and forcing the right upper permanent lateral incisor from its socket, although still retained in the mouth. In this condition he was brought to my office an hour or more after the accident happened. I at once went with him to the office of Dr. Frederick S. McKay, an orthodontist, at that time located in the building on the same floor as myself, and only a few doors removed. When seated the patient presented the following appearance: Right upper lip cut and badly swollen. Right upper temporary cuspid gone (knocked out). Right upper latteral incisor broken into the mouth and remaining in the broken socket with cutting edge presenting toward the soft palate. In trying to bring this tooth to its place in the arch, it was broken from its attachment and fell to the floor. The tooth was bathed in a weak antiseptic solution, the socket syringed out with warm water, the tooth carried to place and retained firmly in the position it had formerly occupied in the arch. The retaining appliance did not interfere with the occlusion of the teeth, was not at all uncomfortable, was absolutely rigid and permitted the syringing and cleansing of the wounded lip and alveolar process. Recoveɹy was rapid. The condition at this time is this: The lateral incisor is firmly in its place in the arch, perfect in color, gum perfect in every feature, the pulp alive and the permanent cuspid just erupting. Drs. Edward H. Angle, G. A. Bowman, H. F. Cassel, Wm. R. Smith, S. H. Voyles, B. E. Lischer and other practicing dentists and orthodontists of St. Louis have seen the case and will testify to the facts as here given, and the patient is living and can be produced at any time to verify the statement made.

I would describe the attachment used in Master Edwin H.'s case as follows: A plain band bearing an "R" tube soldered horizontally was made and cemented to the extracted lateral first. Next, a similar band with tube was made for the firm central, the tubes being placed so as to be in the same horizontal line. A piece of the angle 'G" wire was inserted through both tubes, and the band for the central cemented to place, carrying at the same time the extracted tooth into its socket. The surplus ends of the wire were then clipped, leaving sufficient length to bend up or down distal to each tube. This fixture held the lateral rigidly n the socket until a new union was formed. It remained in place about six months.

I have been thus explicit that it may be established what should and should not be done in similar cases.—February *Denta' Review.*

